# The Advancement and Application of the Single-Cell Transcriptome in Biological and Medical Research

**DOI:** 10.3390/biology13060451

**Published:** 2024-06-19

**Authors:** Kongwei Huang, Yixue Xu, Tong Feng, Hong Lan, Fei Ling, Hai Xiang, Qingyou Liu

**Affiliations:** 1 Guangdong Provincial Key Laboratory of Animal Molecular Design and Precise Breeding, School of Life Science and Engineering, Foshan University, Foshan 528225, China; 2 School of Biology and Biological Engineering, South China University of Technology, Guangzhou 510641, China; 3 Laboratory for Conservation and Utilization of Subtropical Agro-Bioresources, College of Animal Science and Technology, Guangxi University, Nanning 530005, China; aasiax@163.com; 4 Key Laboratory of Molecular Biophysics of the Ministry of Education, Hubei Key Laboratory of Bioinformatics and Molecular Imaging, Center for Artificial Biology, Department of Bioinformatics and Systems Biology, College of Life Science and Technology, Huazhong University of Science and Technology, Wuhan 430074, China

**Keywords:** scRNA-seq, 10 × Genomics, spatial transcriptomics, drug resistance, microbiology

## Abstract

**Simple Summary:**

Single-cell RNA sequencing (scRNA-seq) is a technique that identifies different types of cells in a tissue and reveals how individual cells express genes. This review will examine the evolution of scRNA-seq, which has been widely used in medicine and biology with many results. It will also explore how single-cell transcriptome data can be combined with other omics data such as genome, epigenome, proteome, and metabolome data. The aim of this review is to help scientists to better apply this technology. Finally, this review summarizes the applications of scRNA-seq in cancer, stem cell research, developmental biology, microbiology, and other fields. It also discusses the limitations of this technology and its future developments in biological and medical research.

**Abstract:**

Single-cell RNA sequencing technology (scRNA-seq) has been steadily developing since its inception in 2009. Unlike bulk RNA-seq, scRNA-seq identifies the heterogeneity of tissue cells and reveals gene expression changes in individual cells at the microscopic level. Here, we review the development of scRNA-seq, which has gone through iterations of reverse transcription, in vitro transcription, smart-seq, drop-seq, 10 × Genomics, and spatial single-cell transcriptome technologies. The technology of 10 × Genomics has been widely applied in medicine and biology, producing rich research results. Furthermore, this review presents a summary of the analytical process for single-cell transcriptome data and its integration with other omics analyses, including genomes, epigenomes, proteomes, and metabolomics. The single-cell transcriptome has a wide range of applications in biology and medicine. This review analyzes the applications of scRNA-seq in cancer, stem cell research, developmental biology, microbiology, and other fields. In essence, scRNA-seq provides a means of elucidating gene expression patterns in single cells, thereby offering a valuable tool for scientific research. Nevertheless, the current single-cell transcriptome technology is still imperfect, and this review identifies its shortcomings and anticipates future developments. The objective of this review is to facilitate a deeper comprehension of scRNA-seq technology and its applications in biological and medical research, as well as to identify avenues for its future development in alignment with practical needs.

## 1. Introduction

In recent years, single-cell sequencing has been widely used in biology to investigate the heterogeneity of cell composition in complex organs and the gene expression changes in different cell subpopulations. The advent of scRNA-seq technology has revolutionized our ability to uncover unique gene expression profiles at the individual cell level, facilitating the precise differentiation of various cell types and enabling in-depth molecular mechanism studies. In 2018, Science recognized scRNA-seq technology as the Best Breakthrough Technology of the Year [1]. Since then, it has continuously evolved to meet the demands of various application scenarios. In 2019 and 2020, respectively, Nature Methods acknowledged single-cell multi-omic [2] and spatial transcriptomics [3] as the Technologies of the Year. These technologies are derived from scRNA-seq. In the era of precision medicine, single-cell technologies are expected to play a crucial role in various disciplines.

scRNA-seq is frequently integrated with multi-omics analysis to address biological and medical issues. It has been reported that scRNA-seq has been utilized in conjunction with bulk RNA-seq, genomic, translatomics, proteomic, metabolomic, epigenomic, and other omics data. While the complexity of integrated multi-omics analysis presents a challenge, the advancement of novel analytical techniques will gradually overcome these limitations.

The impact of scRNA-seq technology is extensive, spanning microbiology, neuroscience, developmental biology, immunology, and cancer research. It has been applied to construct large-scale cell atlases [4,5,6,7], refine cell subpopulations and identify rare cell types [8,9,10,11], reveal the evolution and turnover of complex and diverse central nervous system cells [12,13], reveal stem cell development and differentiation trajectories [14,15], and identify microorganisms [16].

This review aims to encapsulate the development trajectory, multi-omics joint analysis, and varied applications of scRNA-seq technology across different fields. Furthermore, this review provides insights into the future directions of this technology. The goal is to offer valuable guidance for its expanded utilization in the realms of biology and medicine.

## 2. Development of the Single Cell Transcriptome

**Figure 1:**
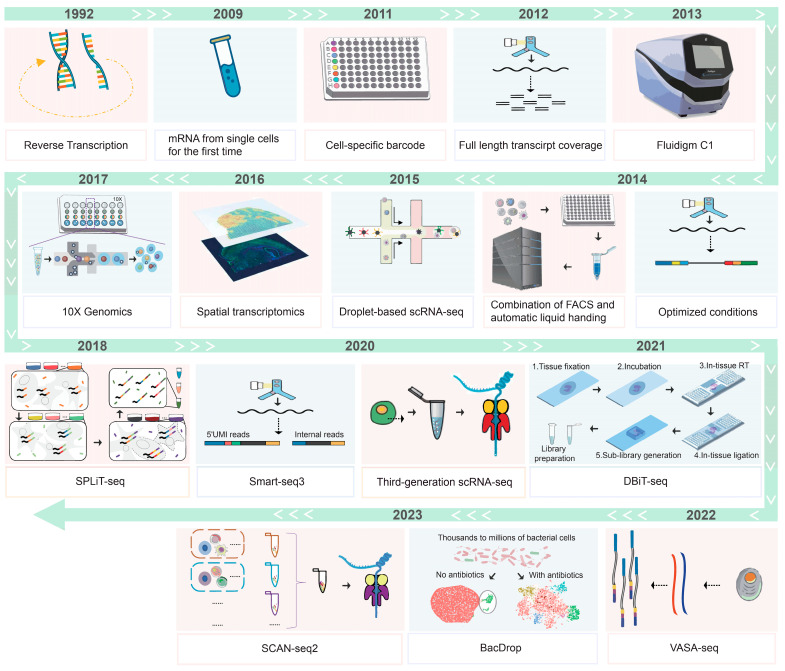
Development of single-cell transcriptome sequencing technology. In 1992, detected gene expression in individual cells using a complex in situ reverse transcription [17]; in 2009, improved the method for microarray analysis, and mRNA was obtained from single cells for the first time [24]; in 2011, barcoding was available for each cell during the reverse transcription process [26]; in 2012, Smart-seq allowed the detection of the full length of mRNA and improved sequence coverage of the transcript [28]; in 2013, Fluidigm C1became the first single-cell automated prep system [29]; in 2013, SMART-seq2 was developed [31]; in 2014, MARS-seq was developed, along with the combination of FACS and automatic liquid handing [32]; in 2015, microfluidics was combined with single-cell RNA-Seq to develop Drop-seq [34] and inDrop [35]; in 2016, spatial transcriptome technology emerged [41]; in 2017, a droplet-based system emerged, the 10 × Genomics Single Cell Transcriptome Sequencing technology [42]; in 2018, the SPLiT-seq was identified through the use of barcode labelling [38]; in 2020, Smart-seq3 combined full-length transcriptome coverage with a 5′ unique molecular identifier RNA counting strategy [43]; SCAN-seq, an scRNA-seq technology based on third-generation sequencing [44]; in 2021, DBiT seq spatial transcriptome technology for simultaneous detection of mRNA and protein emerged [45]; in 2022, VASA-seq exceled in intron coverage and effectively identified non-coding RNA [39]; and in 2023, the technology was updated to SCAN-seq2, which is based on the TGS platform [46].

Single-cell transcriptome sequencing technology has evolved in recent years, and we summarize the research progress of this technology as shown in Figure 1. It started from reverse transcription technology and continues to the latest spatial single-cell transcriptome technology.

In 1992, Eberwine et al. detected gene expression in individual cells using a complex in situ reverse transcription method followed by in vitro transcription amplification [17]. Advancements in PCR methods [18] subsequently led to the increased detection of cells and genes over time [19,20]. By 2003, non-targeted single-cell mRNA amplification techniques enabled transcriptome studies using microarrays [21,22]. In 2009, Tang et al. improved the method for microarray analysis, making it compatible with high-throughput sequencing for an unbiased transcriptome profiling of mRNA from single cells [23,24]. They analyzed mouse embryo blastomeres, showcasing early single-cell sequencing applications for in-depth gene expression analysis in rare cells [23,25]. In 2011, Islam et al. developed a highly multiplexed method capable of barcoding each cell during the reverse transcription process [26]. This method allows a large-scale detection of a variety of mixed-cell samples, such as highly heterogeneous tumor cell samples. Smart-seq, a landmark technology developed in 2012 by scientists in the United States and Sweden, is capable of detecting the full length of mRNA and improving sequence coverage of the transcript. However, it has limitations such as not being strand-specific, having a transcript length bias, and inefficiently transcribing sequences longer than 4 Kb [27,28]. The Fluidigm C1 was commercially introduced in 2013 as the first single-cell automated prep system [29]. A parallel RT-qPCR of over 500 cells for 48 genes identified different cell types without prior classification, enhancing practicality [30]. However, when expanded to tens of thousands of cells or when the goal is to capture as many cells as possible from a limited sample, the scRNA-seq methods described previously meet practical challenges. Conventional microfluidic-based approaches have limited throughput, while plate-based methods typically require time-consuming fluorescence-activated cell sorting (FACS) to separate cells into multiple plates that need to be processed individually. Droplet-based technologies can handle tens of thousands of cells in a single experiment, but current methods require custom microfluidic devices and reagents. The development of methods such as SMART-seq/SMART-seq2 [27,31], MARS-seq [32], STRT-seq [26], and CEL-seq [33] for single-cell transcriptomics has been observed. Harvard University teams have combined microfluidics with single-cell RNA-Seq to develop Drop-seq [34] and inDrop [35] techniques. These techniques provide a rapid and cost-effective analysis of the gene expression of thousands of single cells by encapsulating barcoded beads and cells in droplets for the simultaneous sequencing of all genes.

In 2017, multiple annealing and dC-tailing-based quantitative single-cell RNA-seq (MATQ-seq) was reported by Sheng et al. The MATQ-seq method has been demonstrated to be highly sensitive and to produce a high signal-to-noise ratio in the transcriptome analysis of total single-cell RNA [36]. Subsequently, Homberger et al. refined the MATQ-seq methodology and employed it to investigate the relationship between bacterial transcriptomes and phenotypes in diverse environmental contexts, including infection, persistence, ecology, and biofilm formation [37]. In 2018, Rosenberg et al. developed the SPLiT-seq methodology, which allows for the determination of the cellular origin of RNA by combining barcode labeling. SPLiT-seq is a comprehensive single-cell transcriptome analysis technique that can be applied to complex multicellular systems. Furthermore, SPLiT-seq is compatible with cells or nuclei, allowing for efficient sample multiplexing [38]. In 2022, the vast transcriptome analysis of single cells by dA-tailing (VASA-seq) was developed for comprehensive transcriptome analysis. VASA-seq offers superior coverage of introns and is highly effective in identifying biomarkers of non-coding RNA [39]. In 2023, BacDrop, a method of applying bacteria, was reported to achieve multiple and large-scale parallel sequencing, capable of simultaneously detecting millions of Gram-negative and Gram-positive species. This method is of great significance for the study of bacterial communication and the response of bacteria to disturbances [40].

Spatial localization is crucial for determining cell fate, and in recent years, single-cell spatial transcriptomics techniques have seen rapid development and improvement. Spatial transcriptome technology emerged in 2016 [41]. Among the numerous methods for spatial transcriptomics analysis, the main technologies are divided into two categories, imaging based and sequencing based. In 2017, the 10 × Genomics single-cell transcriptome sequencing technology, was developed to sequence the 3′ end (mRNA) of thousands of single cells [42]. The 10 × Genomics technology, widely utilized in current applications, is characterized by its high throughput and low cost. It can process up to eight samples in parallel and capture approximately 50% of the cells loaded into the system. One advantage of 10 × Genomics is its high cell flux. The microfluidic “double cross” cross system is an 8-channel system, with each channel capable of capturing up to 10,000 cells. Furthermore, the system exhibits high cell adaptability. There are no restrictions on cell size (cell nuclei must be prepared for cells with a diameter exceeding 40 μm) or cell type (tissue cells, immune cells, blood cells, cancer tissue cells, nerve cells, etc.). Nevertheless, the instrument’s shortcomings include its inability to analyze RNA with poly(A) sequences and its requirement for the analysis of large numbers of cells, with the upper range of cells analyzed reaching 10^5^–10^6^. The resolution of spatial transcriptomics is a determining factor in the accuracy of subsequent analysis. Both single-molecule FISH (smFISH) and in situ sequencing provide single-cell resolution [47,48]. Sequential fluorescence in situ hybridization (seqFISH) is derived from smFISH and represents a sequential hybridization-based multiple smFISH method that entails multiple rounds of hybridization, imaging, and probe dissection [49,50]. In each round of hybridization, a set of pre-designed coding probes is employed to detect individual transcripts on multiple occasions for each target. This makes seqFISH highly specific, and its probe design can be used to limit off-target effects. However, the necessity of adding corresponding smFISH probes for each round of hybridization renders seqFISH both expensive and time-consuming [51]. Stereo-seq has a wide field of view and an extremely high resolution. The maximum capture area is 13 cm × 13 cm, and the device exhibits a robust capacity to capture cells. The resolution has been achieved at the nanometer level. Nevertheless, its detection sensitivity is relatively low. The equipment required to operate this device is of a high standard. It is also necessary to optimize the sample processing conditions [52,53]. MERFISH uses a similar combinatorial labeling strategy, using readout probes instead of direct labeling probes [48,54]. The latest versions of these two methods can image mRNA for up to 10,000 genes in individual cells [55]. However, the current application of spatial single-cell transcriptomics is still limited by factors such as cost and expensive detection equipment, making it difficult to be widely adopted on a large scale. In 2021, DBiT-seq was developed for spatial omics sequencing using deterministic barcodes in tissues to construct a multi-omics map on fixed frozen tissue samples. DBiT-seq employs microfluidics to introduce combinatorial DNA oligobarcodes into tissue cells on slices. This technology achieves near single-cell resolution for spatial transcriptomics and targeted proteomics spatial analysis [45]. Here, we conducted a comparative analysis of several spatial transcriptomics technologies, as represented in Table 1. Our objective was to identify the strengths and weaknesses of each technology, thereby providing researchers with a comprehensive reference guide.

Current short-read single-cell RNA sequencing has limitations in analyzing allele and isoform resolution, while long-read sequencing technologies lack sequencing depth. In 2020, Smart-seq3 was introduced, which combines full-length transcriptome coverage with a 5′ unique molecular identifier RNA counting strategy. Compared to Smart-seq2, Smart-seq3 has increased sensitivity, typically detecting thousands of additional transcripts per cell [43]. This is useful for the large-scale identification of cell types and states across tissues and organisms.

In 2020, SCAN-seq was developed as an scRNA-seq technology based on third-generation sequencing (TGS). It is characterized by sequencing read lengths of 1.2 to 2 k bp and 85% accuracy, which was verified by RT-PCR followed by Sanger sequencing [44]. In 2023, the technology was updated to SCAN-seq2, which is based on the TGS platform. This technology has high throughput and sensitivity, allowing for the detection of single-cell full-length transcripts with cDNA lengths up to 1.5–3 k bp before library construction [46]. SCAN-seq2 is a full-length sequencing method that provides uniform transcript information without fragmentation and enrichment. It can be used for the study of a wide variety of biological systems at single-cell and individual RNA isoform resolution and is a valuable tool for the understanding of the complex mechanisms of many diseases.

## 3. Single Cell Isolation and Transcriptome Analysis Process

### 3.1. Single Cell Isolation

The principal methodologies employed in scRNA-seq for the isolation of individual cells include micropipetting micromanipulation, laser capture microdissection, fluorescence-activated cell sorting (FACS), microdroplets, and microfluidics. Micropipetting micromanipulation and laser capture microdissection are techniques employed to isolate a small number of visible cells. These techniques require a significant amount of time. FACS and microfluidics can rapidly separate hundreds of dissociated cells. Microdrops can rapidly separate a large number of cells through fluorescence labeling. Both microdrops and microfluidics technologies can employ FACS for pre-sorting. To summarize, microdrops are currently utilized in an array of single-cell sequencing experiments.

### 3.2. Data Quality Control

Even the most sensitive scRNA-seq protocols generate a small fraction of low-quality barcodes because of lysed or apoptotic cells [56]. To guarantee the reliability of data analysis, data quality control for single-cell transcriptome raw sequencing data is required for subsequent diversity analysis. Cell quality control is typically conducted using three QC factors: the count depth (number of counts per barcode), the number of genes per barcode, and the mitochondrial gene count ratio per barcode [57]. Cell number selection is needed because low-mass cells or empty droplets usually have few genes, double cells may exhibit high gene counts, and low-mass/dying cells usually exhibit extensive mitochondrial contamination. By using the emptyDrops function provided by the DropletUtils package [58], SiftCell [59], dropkick [60], DropletQC [61], and other tools to identify low quality cells or empty droplets. Scrublet [62], DoubletDecon [63], DoubletFinder [64], and other tools recognize two-cell anomalies.

### 3.3. Construction of Seurat Objects and Information Extraction

After filtering the raw data, it is common to use CellRanger to obtain three output files: barcodes.tsv for storing cell information, genes.tsv for storing gene information, and matrix.mtx for the expression matrix [42]. Subsequently, the Seurat object is constructed through Seurat and the data is merged. Seurat is an R package designed for cellular quality control and analysis of single-cell RNA-seq data, capable of identifying and interpreting sources of heterogeneity in single-cell transcriptome data, as well as providing functions to integrate different types of single-cell data [65]. Overall, Seurat performs single-cell dataset preprocessing, the computation of typical correlation vectors, the identification of rare non-overlapping subpopulations, and the alignment of typical correlation vectors, and integrates analysis across datasets.

### 3.4. Cluster Analysis

As single-cell transcriptome sequencing data have multiple covariances, there are inter-correlations between feature attributes, which can lead to a spatial instability of the solution, thus resulting in a weak generalization ability of the model. The high-dimensional spatial samples have sparsity, which causes the model to be more difficult to find in the data features. Therefore, dimensionality reduction analysis is necessary for data processing to find the essential structural features within the data through dimensionality reduction algorithms, such as feature selection or feature extraction. Cluster analysis groups data objects based on their similarity to each other. Common clustering methods used are Principal Components Analysis (PCA), t-distributed Stochastic Neighbor Embedding (t-SNE), and Uniform Manifold Approximation and Projection (UMAP). PCA is a technique for analyzing and simplifying data sets by mapping the data from the original vector space to a new space [66]. t-SNE is capable of mapping data in a high-dimensional space into a low-dimensional space and preserving the localized characteristics of the dataset [67]. UMAP is a novel method for dimensionality reduction in manifold learning, founded on Riemannian geometry and algebraic topology theory [68].

### 3.5. Annotation of Cellular Taxa

Cellular annotation is a significant part of single-cell functional analysis, and the information on cellular subpopulations as to specific cell types (including subtypes) does provide a central reference for subsequent data analysis and molecular mechanism revelation. The general workflow of cellular annotation in single-cell transcriptome data analysis, is from sequencing data to generating a complete cellular subpopulation map with annotations. There are three main steps in the analysis process: automated annotation, manual annotation, and validation [69].

#### 3.5.1. Automatic Annotation

Automated cellular annotation methods are used to annotate cells by comparing the data to annotated reference data, or by using known marker genes for a particular cell type. Automated annotation uses a set of predefined “marker genes” (e.g., genes specifically expressed in known cell types) or reference single-cell transcriptome data (professionally annotated single-cell atlases) to identify and label individual cells or clusters of cells by matching their gene expression patterns to those of known cell types. There are two main methods of automated annotation: one uses known marker genes, and known relationships between marker genes and cell types are available from databases, such as PanglaoDB [70] and CellMarker [71]. The other approach is to compare the scRNA-seq data to be annotated (the “query” dataset) with an existing, similar, professionally annotated scRNA-seq reference dataset, the “reference” dataset being derived from GEO [72], single-cell expression profiles, cellular annotation profiles [73], etc.

#### 3.5.2. Manual Annotation

Although the automated cell annotation method is convenient and systematic, it necessitates suitable reference databases, and the annotation outcomes could be of low confidence. When automated annotation results in low confidence, anomalous cell type proportions, or omissions, specialized manual annotation is required. In manual cellular annotation, individual cell expression is analyzed for function clues using the same principles as marker-based automated annotation. Professional manual annotation is frequently considered the standard level of rigor in cellular annotation; nonetheless, the annotation process is time-consuming, laborious, and involves some subjectivity.

#### 3.5.3. Validation

Automated and manual annotation can provide reliable cell type labeling for scRNA-seq data. In practice, mRNA-based assays can only partially define cell type and function, and confirmation of new cell types must be experimentally validated to increase accuracy and confidence. For example, this could be performed using T-cell receptor (TCR) [74] and B cell receptor (BCR) [75] clonotyping to refine the type of immune cells in the tissue. At the same time, annotated cell types are co-validated with the help of single-cell ATAC-seq and the spatial transcriptome [47]. In addition, tumor tissues can be analyzed in conjunction with scRNA-seq data for copy number variants (CNVs), where changes in CNVs result in consistent up-regulated (amplification events) or down-regulated (deletion events) expression changes of consecutive genes on the genome [76].

### 3.6. Calculation of Cell Ratios

After classifying cell taxa in the single-cell transcriptome, the discussion of the function, development and differentiation of different cell subtypes and the possibility of obtaining precise cell subtypes requires calculations of the ratio of the number of cells, especially in immunological studies, where changes in the ratio of immune cells in different populations are of great interest.

Crude cell scale formula: P(d) = 1 − (1 − s) n [77] (P(d): power of detection; s: proportion of target cell subpopulations; and n: number of cells to be sequenced)

However, this preliminary calculation method does not consider the necessary number of cells replicates to decrease false positive and false negative error rates and eliminate random errors, which generally require 2–5 replicates.

In contrast to the crude cell ratio, the Seurat v3 [78] (https://satijalab.org/seurat/, accessed on 31 May 2019) number estimation method considers not only the minimum frequency cell subpopulation, detection power, but also the number of possible cell subpopulations and the minimum cell detection. The Satija lab has developed a web tool [65] (https://satijalab.org/howmanycells, accessed on 15 April 2015) for this algorithm, which adjusts parameters to calculate the number of cells needed for sequencing.

### 3.7. Analysis of Cellular Interactions

Intercellular communication coordinates complex organic processes in living organisms that require the synergy of multiple cell types. The dysregulation of autocrine signaling is an example of defects in intercellular communication that is also important in cancer, autoimmune, and metabolic disease research [79]. To examine the cellular communication networks amidst dissimilar cell types during physiological processes, the Teichmann Lab and the Vento-Tormo Lab at the Sanger Institute in the UK have collaboratively created CellPhoneDB [80] (https://www.cellphonedb.org/, accessed on 14 November 2018) for the scrutiny of single-cell transcriptome interactions. CellPhoneDB software analyzes single-cell transcriptome data by using known protein interaction annotations. It generates visual heat maps to investigate interactions and communication mechanisms in living systems at a precise cellular level. However, the results offered by CellPhoneDB cannot be visually presented directly. The iTALK software (https://italk.sourceforge.net/) can display the cell-type interaction network of CellPhoneDB outcomes. It can facilitate comparing differences between groups, drawing intercellular interactions, and creating chordal diagrams of receptor and ligand relationships.

### 3.8. Gene Differential Expression Analysis

Single-cell transcriptome difference analysis is comparable to transcriptome analysis and typically utilizes DESeq [81] and edgeR [82] to detect genes that are differentially expressed. First, the fold change is calculated to determine the differences in gene expression between sample groups. Then, the significance of these gene expression differences is determined using a difference test and *p*-values are obtained. The standard DESeq algorithm is commonly used for transcriptome studies involving biological duplicates to identify differentially expressed genes. For studies without biological replicates, the TMM standard method in edgeR can be utilized to identify differential gene expression. Following cell colonization, differentially expressed genes (DEGs) are classified and compared using the FingMarkers function. The iDEA tool, which combines differential expression and gene set enrichment analyses, can enhance analysis efficiency by utilizing gene information based on DEG effect size distribution properties and modeling all genes collectively within a joint statistical framework. iDEA has been introduced as an effective approach for such purposes [83].

### 3.9. Pseudotime Analysis

Throughout life, cells respond to stimuli by transitioning between functional states that express different genes. This process involves the transcriptional reorganization of genes, whereby some are silenced and others activated. However, purifying these transient cells for research purposes proves nearly impossible. To overcome this issue, researchers utilize single-cell transcriptome sequencing through the pseudotime analysis method [84]. Monocle, a tool for the pseudotime analysis of single-cell RNA sequencing, applies algorithms to identify changes in gene expression during cellular state transitions and determine differentially expressed genes by pseudotime values through the differential Gene Test function [85]. Branching nodes may signify cells undergoing programmed changes, such as cell fate differentiation, which underscores the significance of analyzing branching events. The BEAM (Branched Expression Analysis Modeling) method is frequently employed to analyze the cell data’s nodes post the suggested temporal ordering.

## 4. Integrated Analysis of scRNA-Seq and Multi-Omics

**Figure 2:**
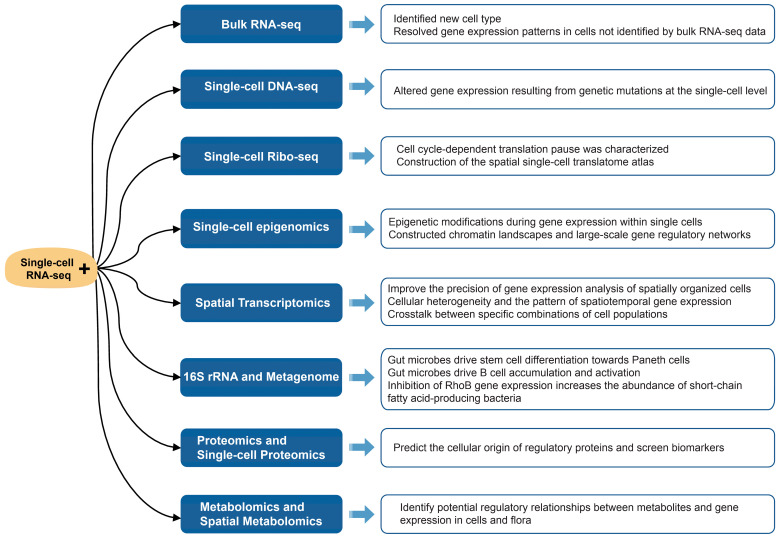
Combined applications and advantages of scRNA-seq and multi-omics.

Single-cell RNA sequencing (scRNA-seq) is an effective technique for the identification of cellular subpopulations within tissues, enabling researchers to conduct highly precise studies of the epigenetic modification, transcription, translation, and spatial distribution of genes in cells. In this section, we present a summary of the applications of the single-cell transcriptome with bulk RNA-seq, single-cell genomics, microbiomics, metabolomics, and other multi-omics integrated analyses (as shown in Figure 2). This is done with the intention of providing novel ideas for research in biology and medicine.

### 4.1. Bulk RNA-Seq

Bulk RNA-seq is a cost-effective method commonly used to analyze gene expression in a large number of cells. However, it cannot capture the heterogeneity of different cell types within tissues. To overcome this limitation, single-cell RNA-seq (scRNA-seq) is employed to analyze the gene expression of individual cells. The integration of both approaches allows for a more comprehensive understanding of the biological functions of differentiated tissue cells.

In order to resolve the gene expression profiles of mouse and human dendritic cells, new dendritic cell subtypes (T-bet-cDC2s and T-bet+cDC2) were identified by scRNA-seq, bulk RNA-Seq and ATAC-Seq, revealing the transcriptional basis of mouse and human dendritic cell heterogeneity [86]. The study aimed to investigate the effects of age on lung cells. It found that increased cholesterol synthesis in type 2 pneumocytes and adipose fibroblasts, as well as alterations in respiratory epithelial cells, are hallmarks of lung aging. This was revealed by bulk RNA-seq, scRNA-seq and protein profiling. Additionally, the study demonstrated that lung cells of senescent mice exhibit increased transcriptional noise and decreased rigor in the control of epigenetic regulation [87]. To elucidate the role of distinct cell types within skeletal muscle on fat deposition, myeloid-derived cells (52.13%), fibroblast/fibro/adipogenic progenitors (FAPs) (23.24%), and skeletal muscle stem cells (2.02%) were investigated. This study reveals that a subpopulation of myeloid-derived cells may contribute to intramuscular fat infiltration [88].

These studies identified new cell types, including dendritic cells, type 2 pneumocytes, and myeloid-derived cells by an integrated analysis of scRNA-seq and bulk RNA-seq, and resolved gene expression patterns in cells not identified by bulk RNA-seq data.

### 4.2. Single-Cell DNA-Seq

Single-cell DNA sequencing (scDNA-seq) is a method that identifies intercellular heterogeneity and analyzes genetic maps at the cellular level by sequencing single-cell genomes. The integrated analysis of scRNA-seq and scDNA-seq technologies reveals altered gene expression due to genetic differences in individual cells.

The development and progression of cancers is driven by the accumulation of mutations. The differential analysis of cancer cells and paraneoplastic tissues in patients is a valuable approach for cancer treatment and drug discovery. G&T-seq is a method for the simultaneous sequencing of single-cell genomes and transcriptomes [89], which is a powerful tool for studying cellular mutations that cause disease. To gain insight into the evolution of copy number alterations in hepatocellular carcinoma, the common clonal origin of diploid and polyploid aneuploid cells were identified by scDNA-seq and scRNA-seq. It was demonstrated that polyploid tumor cells are generated by a whole genome doubling of diploid tumor cells [90]. The evolution of breast cancer was found to occur intermittently based on analyses of cancer cell copy number changes in the breast, as revealed by scDNA-seq and scRNA-seq [91]. To investigate the phenomenon of cytogenetic rescue, the clinical evolution, genetic features, and trajectories of myelodysplastic syndromes caused by SAMD9/9L mutations were revealed by the combined analysis of scDNA-seq and scRNA-seq [92]. The combined analysis of the scDNA-seq and single nucleus RNA sequencing (snRNA-seq) of brain tissue from children with autism revealed that synaptic function, nerve growth, and migration were affected in the neocortex of the patients’ brains. Furthermore, a specific set of genes enriched in upper projection neurons and microglia demonstrated a correlation with clinical severity in patient samples [93].

The integration of scRNA-seq and scDNA-seq enables the investigation of altered gene expression resulting from genetic mutations at the single-cell level. This approach offers a novel perspective for precision medicine and the development of cancer drugs.

### 4.3. Single-Cell Ribo-Seq

Ribo-seq is a technique for detecting the translation of transcripts. In 2021, scRibo-seq was reported to measure translated transcripts with single codon resolution, and cell cycle-dependent translation pause was characterized by this technique [94]. In 2023, scRibo-seq was coupled with the spatial transcriptome, resulting in the construction of the spatial single-cell translatome atlas [95]. Fewer scRibo-seq studies have been reported, and the future application of this technique may reveal more individual cell translation regulation mechanisms.

### 4.4. Single-Cell Epigenomics

Single-cell epigenomics techniques include the single-cell assay for targeting accessible-chromatin with high-throughput sequencing (scATAC-seq), single-cell methylated RNA immunoprecipitation sequencing (scMeRIP-seq), and scDNA methylation sequencing, etc. These technologies, when used in conjunction with scRNA-seq analysis, reveal the regulatory role of epigenetic modifications during gene expression within single cells.

To explore the role of m6A modification on the tumor immune microenvironment, colorectal cancer (CRC) tissues were examined by scRNA-seq and MeRIP-seq, ribo-seq, and bulk RNA-seq techniques. The study revealed that YTHDF1 impairs anti-tumor immunity to promote CRC through the m6A-p65-CXCL1/CXCR2 axis, which could be a potential target for therapy [96].

A joint analysis of scRNA-seq and scATAC-seq data quantified statistically significant correlations between anticodon usage and amino acid supply in mouse and human single-cell ATAC-seq profiles. In comparison to other cell types, neurons exhibited enhanced translation efficiency, driven by an augmented supply of tRNA-Ala (AGC) peptides. This suggests that reductions in tRNA-Ala (AGC) anticodon libraries may be pertinent to neuropathology [97]. In skeletal muscle, scRNA-seq and scATAC-seq revealed an increase in TNNT2+ and DCLK1+ muscle stem cells and a significant decrease in ENOX1+ muscle stem cells during aging [98]. Zhang and colleagues constructed accessible chromatin landscapes and large-scale gene regulatory networks for human, monkey, and mouse cells using scATAC-seq and scRNA-seq [99]. Several methods have been developed for the integrated analysis of scATAC-seq and scRNA-seq [100,101,102].

In a combined scRNA-seq and single-cell methylome analysis, Johnson and colleagues integrated data from 914 single-cell DNA methylomes and 55,284 scRNA-seq. Their findings indicated that localized DNA methylation impairments in gliomas were associated with cell-to-cell differences in DNA methylation, which were elevated in more aggressive tumors [103]. Hou et al. developed scTrio-seq, a single-cell genome, DNA methylome, and transcriptome sequencing technology. This technology was applied to 25 single cancer cells derived from human hepatocellular carcinoma tissue samples, resulting in the identification of two subpopulations of cells that were identified based on CNVs, DNA methylome, and transcriptome analysis [104].

It is noteworthy that, despite the availability of new sequencing methods that are simultaneously compatible with multiple single-cell epigenomics, the combined simultaneous application of the multi-omics to the same individual cell remains a challenge.

### 4.5. Spatial Transcriptomics

Although Spatial Transcriptomics (ST-seq) can obtain both spatial location information and the gene expression data of cells, it currently falls short of single-cell precision. The integration of scRNA-seq and ST-seq offers a promising avenue for enhancing the precision of spatial cellular gene expression analysis.

Cardiac cells are complex and diverse, and their functions and gene expression need to be further revealed. The cellular heterogeneity of different parts of the heart and the pattern of spatiotemporal gene expression during cardiac development were investigated by scRNA-seq and ST-seq to construct an expression map of the developing heart. The upper region of the heart has higher cellular diversity, and gene expression differences between different regions of the heart are more pronounced than those between time points [105]. The techniques of scRNA-seq and MERFISH were integrated to analyze spatial information and identify a wide range of cardiomyocytes. This approach resulted in the creation of a unique structure of the human heart, including previously uncharacterized cardiac cell populations. Additionally, it revealed signaling pathways that coordinate intercommunication between cardiac cell populations that form this structure. The examination of crosstalk between specific combinations of cell populations in these communities revealed differential signaling pathways, including the organic protein-hemosporin that directs multicellular interactions during ventricular wall morphogenesis [106].

The world’s inaugural spatial single-cell atlas of the whole brain cortex of a non-human primate was created by a coronal sectioning of the left hemisphere of the brain of adult male crab-eating macaques. This approach combined spatial and scRNA-seq, identified 264 cell types, and mapped their spatial distribution throughout the cortex [107]. To elucidate the mechanisms of functional decline during cellular senescence in the brain, scRNA-seq combined with ST-seq were used to construct high-resolution cellular maps of brain senescence in the frontal cortex and striatum. The researchers observed that cell state, gene expression, and spatial organization were more pronounced in non-neuronal cells than in neurons. The activation of neuroglial and immune cells during aging was enriched in subcortical white matter, and similarities and significant differences in cellular activation patterns induced by aging and systemic inflammation were identified. These findings provide important insights into age-related decline and inflammation in the brain [108].

To study the development of the embryonic lung, the authors first investigated the gene expression patterns of proximal and distal epithelial cells in the early embryonic lung with the help of scRNA-seq, then studied the ecological niche of the proximal and distal lung in the early embryonic lung by ST-seq, and finally deepened the understanding of the proximal–distal pattern of the epithelium in the development of the human lung. The combination of the two groups led to a deeper understanding of this pattern, opening new avenues for regenerative medicine [109].

The integration of scRNA-seq analysis with ST-seq offers a highly effective and precise approach to elucidating the intricate cellular processes occurring within complex organs, such as the heart and brain. Moreover, the combination of these two technologies provides invaluable insights into the communication and coordination of diverse cell types during organogenesis.

### 4.6. 16S rRNA and Metagenome

The integration of 16S rRNA-seq, metagenome, and scRNA-seq analyses is a valuable approach for uncovering the biological functions of microorganisms and identifying microbial–host interactions.

The interaction of gut microbes with host inflammation and immunity has been a subject of intense research in recent years. Kim and colleagues demonstrated that gut bacteria promote stem cell differentiation through an scRNA-seq and 16S rRNA-seq. Following antibiotic treatment during early childhood, an impaired differentiation of stem cells to Paneth cells and macrophages was observed through scRNA-seq, and a reduction in the number of Paneth cells was linked to necrotizing enterocolitis (NEC). 16S rRNA-seq revealed a significant decrease in the number of lactobacilli in the NEC model mice. However, lactobacilli colonization promoted Paneth cell differentiation and reduced the severity of NEC. The results suggest that gut microbes (Lactobacillus) drive stem cell differentiation to Paneth cells through macrophage and mesenchymal cell ecological niches to limit NEC [110]. Yang and colleagues employed scRNA-seq and 16S rRNA-seq of RhoB genes to investigate the influence of gut microbes on colitis development. Their findings revealed that the RhoB gene was highly expressed in colonic tissues of colitis model mice. Additionally, they demonstrated that RhoB gene defects could ameliorate the symptoms of dextran sulphate sodium (DSS)-induced acute and chronic colitis. 16S rRNA-seq revealed a significant increase in the abundance of short-chain fatty acid-producing flora (Prevotella and Alloprevotella) that inhibit DSS-colitis. Furthermore, enterobacteria from RhoB gene-deficient mice exhibited higher resistance to colitis. These findings suggest that the inhibition of RhoB gene expression may increase the abundance of short-chain fatty acid-producing bacteria and thus inhibit the development of colitis [111]. Helicobacter hepaticus (Hhep) is an adherent bacterium found predominantly in the cecum and colon that induces a local immune response. Delgoffe and colleagues found that Hhep-CRC mice inhibited tumor growth using scRNA-seq. Hhep colonization induced Hhep-specific Tfh cells, increasing the number of colonic Tfh, and the introduction of the immunogenic gut bacterium Hhep enhanced the anti-tumor immune effect in CRC [112].

The regulatory axis of gut microbes and organogenesis also plays a role in modulating disease development. Barrow and colleagues reveal potential mechanisms by which microorganisms exacerbate fatty liver. B-cell enrichment and a high expression of pro-inflammatory genes in the livers of mice modeling non-alcoholic steatohepatitis (NASH) were identified by scRNA-seq, and 16S rRNA-seq revealed that the fecal transplantation of patients with non-alcoholic fatty liver disease induced changes in the intestinal microbial composition of the recipients, as well as the accumulation and activation of intrahepatic B-cells, which contributed to the progression of NASH. These findings indicate that gut microbes drive B cell accumulation and activation, which in turn promote hepatic inflammation and fibrosis [113]. The integration of scRNA-seq, 16S rRNA-seq, and macrogenomic sequencing has enabled the identification of gut microbes regulating chronic obstructive pulmonary disease (COPD) through the gut–lung axis. The intestinal flora was found to be associated with the pathogenesis of COPD by 16S rRNA-seq and macrogenomic sequencing. Parabacteroides goldsteinii (Pg), a differential bacterium that was negatively associated with COPD severity, was identified. In addition, MTS01 treatment was found to alter the cellular composition and function of intestinal and lung tissues and to enhance the ribosomal and mitochondrial activity of intestinal cells, as demonstrated by scRNA-seq analysis. The results indicate that Pg and its products may be employed as alternative pharmaceutical agents for the prevention or treatment of COPD [114]. Probiotics are not always beneficial for the host, and lactobacilli may contribute to the development of pancreatic cancer. Hezaveh and colleagues observed that the gene for the aromatic hydrocarbon receptor (AhR), Cyp1b1, was significantly upregulated in patients with pancreatic ductal carcinoma using bulk RNA-seq. They also found that inhibiting AhR expression enhanced the anti-tumor effect. Three related clusters of macrophages were identified in tumor tissues by scRNA-seq. The results of 16S rRNA-seq demonstrated that antibiotic treatment altered the structure of the intestinal flora, revealing that lactobacilli activated tumor-associated macrophage AhR, which exerted an inhibitory mechanism to promote the growth of pancreatic cancer and destroy the immune checkpoints [115].

Rumen microbes are closely related to the host’s digestion of food for nutrient acquisition. Sun et al. employed macrogenomes to identify rumen flora that metabolize cellulose and then explored epithelial cell taxa that take up these metabolized fatty acids through scRNA-seq methods. This study identified key individual microbial genomes and epithelial cell subtypes in the rumen that are involved in fiber digestion, VFA uptake, and metabolism, respectively. The microbial genome and epithelial monocytes were linked to the nutrient system by macrogenomic and scRNA-seq integration analyses [116].

Ghaddar and colleagues developed a single-cell analysis of host-microbe interactions that identifies microbial information from sequences of host tissue scRNA-seq. The application of this method to pancreatic cancer studies revealed that microbes were present in the majority of tumor cells and virtually absent in normal tissue cells. Tumor microbes significantly affected host cell gene expression and activated tumor immune responses, and tumor microbes predicted lower survival [117].

The current combination of scRNA-seq, 16S rRNA-seq and macrogenomics has revealed the association of microbes with host immunity, disease, development, and nutrition.

### 4.7. Proteomics and Single-Cell Proteomics

The association of proteomics and bulk RNA-seq has been well-established, and studies combining proteomics and scRNA-seq have gradually emerged in recent years. In contrast, the combination of proteomics and scRNA-seq elucidates more information at the level of a single cell or a certain type of cell, which helps researchers to predict the cellular origin of regulatory proteins and screen biomarkers.

The combination of scRNA-seq and proteomics can be used to identify biomarkers from body fluids such as plasma and urine. Chan et al. screened 83 biomarkers of heart failure after myocardial infarction by combining the plasma proteome and scRNA-seq, of which four were newly identified: ANGPT2, THBS2, LTBP4, and FSTL3. The question of cellular origin corresponding to the markers based on scRNA-seq data was revealed [118]. Urinary proteins reflect immune activation profiles and thus assist in the classification of patients with lupus nephritis. The urine proteome was employed to stratify patients on a gradient of IFN-γ-induced chemokines, thereby identifying those with proliferative lupus nephritis. The scRNA-seq was used to identify the cellular source of the chemokines detected in urine [119].

Pancreatic ductal adenocarcinoma is a fatal disease with limited therapeutic options and a low survival rate. Zhou and colleagues used spatial transcriptome, snRNA-seq, bulk RNA-seq, and proteomics to distinguish pancreatic tumor cells from normal and transitional cells. The tumor and transitional cell subpopulations were analyzed by pathology-assisted spatial transcriptome data [120]. To investigate the fate and functional mechanisms of pancreatic islet endocrine subpopulations in health and disease, scRNA-seq was combined with single-cell proteomics analysis to elucidate the functional status of each α-, β-, and δ-cell subpopulation in glucose intolerance. The ACE2, CD81, and GLUT2 markers screened in the study enable the differentiation of α, β, and δ cell subpopulations based on their maturity and functional status. Targeting these markers may alter islet subpopulation distribution, thereby enhancing islet function [121]. Multiple myeloma, which is prone to drug-resistant relapse, is an adult hematologic malignancy. Yao and colleagues conducted a study in which they analyzed bone marrow samples from 41 patients using scRNA-seq analysis, followed by bulk RNA-seq and proteomic cross-validation of the expression of candidate targets. The objective of this study was to identify potential therapeutic targets for multiple myeloma [122].

The integration of scRNA-seq with proteomics, or single-cell proteomics, has been employed in the analysis of biological samples, including blood, urine, bone marrow, and organelle assays. This approach has proven effective in the identification of biomarkers and cellular subpopulations, and it has emerged as a valuable tool for the discovery of new drugs and the development of novel therapeutic strategies for various diseases.

### 4.8. Metabolomics and Spatial Metabolomics

Metabolomics, single-cell metabolomics, and scRNA-seq can be used in an integrated analysis to identify potential regulatory relationships between metabolites and gene expression in cells and flora. This tool is a valuable resource for elucidating the impact of intestinal substrates on the structure of the flora, and the influence of the tumor microenvironment on cancer, organ development, and disease markers.

The TMAO-induced immune activation phenotype was found to correlate with the activity of the gut flora enzyme CutC/D by metabolomic testing of serum samples from a PDAC mouse model. Subsequently, the authors conducted further analysis of the immune cells using bulk RNA-seq and scRNA-seq, which revealed that TMAO directly promotes the transition of tumor immunosuppression to an immune activation state. This finding elucidates the relationship between the intestinal flora-derived metabolite TMAO and the anti-tumor immune response [123].

Intrahepatic cholangiocarcinoma (ICC) is a rare malignant tumor. Previous studies have suggested that microorganisms within tumors can independently produce anticancer effects, and that different types of tumors even have their own unique community structures. However, the specific biological functions of bacteria still need to be further investigated. Chai and colleagues conducted a study to analyze the microbiota characteristics of intrahepatic cholangiocarcinoma. This was done by using scRNA-seq in conjunction with tissue metabolome and cell transcriptome analysis. The results revealed that Umbelliferae spp. in paracancerous tissues were inhibiting tumor growth, suggesting that the bacteria in ICC may play an anti-tumor function [124]. Inflammatory bowel disease (IBD) is a term that encompasses two main conditions: Crohn’s disease and ulcerative colitis. The colonic immune microenvironment and underlying regulatory mechanisms of individuals with IBD have not been well studied. Here, the authors examined the immune profile and metabolic microenvironment of untreated individuals with IBD by single-cell transcriptomic, metabolomic, bulk RNA-seq, and mass spectrometry analyses. Selenium was found to act as an important regulator of T cell responses and a potential therapeutic target for celiac disease [125]. Obesity represents a significant risk factor for cancer, yet the precise manner in which differences in systemic metabolism affect the tumor microenvironment (TME) and influence anti-tumor immunity remains unclear. In this study, the authors constructed a single-cell resolution map of cellular metabolism by integrating bulk RNA-seq, scRNA-seq, lipidomics, metabolomics, and TMT-proteomics multi-omics association. In obese mice, the blockade of the metabolic reprogramming of tumor cells has been demonstrated to improve anti-tumor immunity. The study demonstrated that a high-fat diet induced obesity impaired CD8+ T cell function and accelerated tumor growth in the mouse TME [126]. Pancreatic ductal adenocarcinoma (PDAC) is a highly lethal cancer that frequently demonstrates resistance to chemotherapy. Tumor-associated macrophages (TAM) play a pivotal role in regulating the TME, including the promotion of chemotherapeutic resistance. In their study, Zhang and colleagues analyzed chemotherapy-treated samples from humans and mice using a multi-omics approach, which included scRNA-seq, bulk RNA-seq and metabolomics. Four major TAM subpopulations in PDAC were identified, with proliferating resident macrophages (rMφs) being strongly associated with poor clinical outcomes. Targeting proliferating rMφs may represent a potential therapeutic strategy for enhanced chemotherapy in PDAC [127].

Metabolism is recognized as a key driver of cellular differentiation during development. It is of great importance to investigate the potential metabolic heterogeneity and complexity during human kidney development. Rabelink and colleagues identified metabolic cell fate trajectories in human kidney differentiation using scRNA-seq, spatial metabolomics at the single-cell level combined with immunofluorescence [128].

By employing scRNA-seq, spatial transcriptome, and AFADESI spatial metabolomic technology, Zheng and colleagues analyzed brain samples from patients with traumatic brain injury and characterized genes of the lipid peroxidation subgroup as being associated with neuronal loss in damaged brain regions. The study identified imbalanced inositol and inositol phosphates and associated spatial markers in the lipid peroxidation region of injured neurons [129].

The metabolome is a key factor in understanding cellular physiological processes, as it resolves the end products of cellular physiological activities. This is particularly important in conjunction with scRNA-seq, which is a valuable tool for elucidating cellular physiological processes. The current emergence of single-cell metabolomic and spatial metabolomic technologies will gradually enable the functional resolution of tissue in situ cells.

## 5. Application of Single-Cell Transcriptome Sequencing Technology

**Figure 3:**
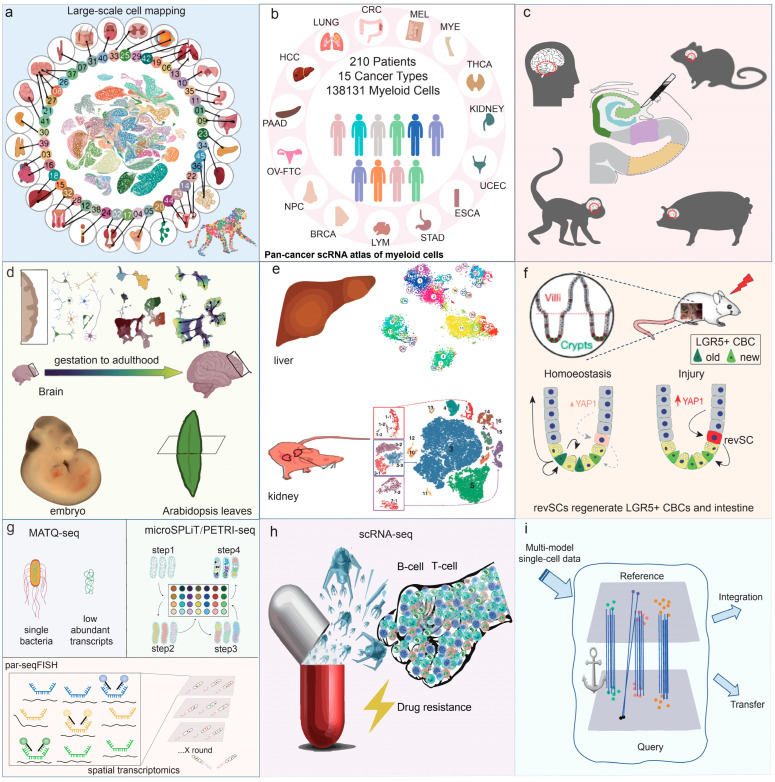
Applications of single-cell transcriptome sequencing in different fields. (**a**) Large-scale cell mapping construction, the first whole-body organellar transcriptome mapping in a non-human primate, the rhesus macaque [7]. (**b**) Oncology research: pan-cancer analyses on bone marrow cells from 210 patients with 15 different human cancer types using single-cell transcriptome sequencing [130]. (**c**) Neuroscience research: hippocampus of humans, rhesus monkeys, and pigs [12]. (**d**) Developmental biology: human prefrontal cortex gene expression from gestation to adulthood [131], embryo and Arabidopsis leaves [132]. (**e**) Cell subpopulation refinement and rare cell type identification: liver [10] and kidney [11]. (**f**) Stem cells research: resurrection stem cells in the mouse intestine, which can be activated through injury [15]. (**g**) Applications in microbiology: protocols for bacterial single-cell transcriptomics [133]. (**h**). Single-cell transcriptome reveals drug resistance in cancer cells. (**i**) Integration and utilization of single-cell datasets: “anchor” different datasets together, which not only integrates single-cell sequencing data with different scRNA-seq technologies, but also integrates single-cell sequencing data with different modalities [78].

As shown in Figure 3, scRNA-seq plays a great role in the fields of cellular atlas construction, organ development, disease research and brain science research. In this section, we summarize the applications of single cells in research and list some representative works.

### 5.1. Large-Scale Cell Mapping Construction

The construction of large-scale transcriptomic maps of cells is pivotal in elucidating the biological mechanisms associated with health and disease. Single-cell transcriptomics facilitates the identification of gene expression patterns and regulatory networks in cells of tissues and organs.

In 2020, Dong et al. mapped the single-cell transcriptome of 28 immunophenotypic blood cell populations in the steady-state hematopoietic system of mice. They were able to decipher the changing law of hematopoietic reconstruction kinetics in the early stage of hematopoietic stem cell transplantation in mice [4]. In the same year, Xie et al. comprehensively mapped transcriptome profiles of 32 different human blood cell types and their corresponding regulatory profiles for transcription factors. An online search platform was constructed for predicting blood cell types and functions and analyzing gene expression. This platform provides an essential transcriptome reference for extensive research on blood cells around the globe [5]. In 2021, Zhang et al. systematically mapped the single-cell transcriptome profiles of primate hippocampal aging. The article presents the revealing of the molecular mechanism causing hippocampal hypoplasia during aging. This discovery forms a basis for identifying early warning markers and potential intervention for hippocampal senescence and related degenerative diseases [6]. In 2022, Han et al. conducted the first whole-body organellar transcriptome mapping in a non-human primate, the rhesus macaque. This atlas will serve as a crucial resource for research in species evolution, human diseases, and drug evaluation and screening. It will provide essential tools for developing biomedical resources and aid in disease diagnosis and treatment. It will also aid in targeted drug development and provide opportunities for a more thorough exploration of life evolution [7].

### 5.2. Oncology Research

ScRNA-seq plays a vital role in mapping cancer cells, characterizing malignant cell heterogeneity, profiling tumor gene expression, identifying new cell subpopulations, and exploring immune cell heterogeneity and the immune microenvironment [134]. Researchers have utilized scRNA-seq to analyze the diverse landscape and tumor microenvironment composition of distal cholangiocarcinoma in humans [135]. A cancer cell subtype, which departs from the typical differentiation trajectory and dominates metastasis, has been detected in the normal tissues of lung cancer patients, as well as in early to metastatic cancer cells. This study enhances our comprehension of the molecular and cellular dynamics involved in metastatic lung cancer, identifying potential therapeutic targets in cancer–microenvironment interactions [136]. Cheng and colleagues conducted pan-cancer analyses on bone marrow cells from 210 patients with 15 different human cancer types using scRNA-seq. This study revealed varying attributes of myeloid cells infiltrating tumors of diverse cancer types, indicating promising prospects for targeted immunotherapy [130]. The heterogeneous properties of tumor tissues make them amenable to scRNA-seq, and alterations in cancer cell profiles frequently anticipate oncogenic modifications in the same tissues. Changes in cancer cell transcriptional levels allow for the timely monitoring of tumor formation and metastasis, establishing a molecular reference point for physicians when treating patients.

### 5.3. Neuroscience Research

Franjic and colleagues conducted an analysis of single-cell transcriptome integration within the hippocampus of humans, rhesus monkeys, and pigs. They identified a unique trajectory of the cellular differentiation of hippocampal granule cells, from neural stem cells to mature granule cells. However, this neural pathway is absent in the human brain [12]. Wang et al. examined the temporal pattern of neuropathic pain by analyzing single-cell RNA sequencing (10 × Genomic) of the dorsal root ganglion in mice with nerve injury-induced pain. This study’s findings provide novel insights into comprehending the dynamic process of neuronal type alterations and their associated molecular mechanisms during the progression of neuropathic pain [13]. These studies demonstrate that utilizing single-cell transcriptome technology in neuroscience research helps to unveil the evolution and turnover of complex and diverse cells within the central nervous system, providing numerous benefits.

### 5.4. Developmental Biology

In investigations of brain development, scRNA-seq techniques have mapped complex brain cell gene expression by distinguishing brain cell populations. Kanton et al. analyzed temporal cellular maps of brain development in chimpanzees and rhesus monkeys using scRNA-seq and accessible chromatin profiling. Human-specific dynamic gene regulatory features were discovered [137]. Raj and colleagues created a single-cell catalog for zebrafish brain development, consisting of approximately 220,000 cells across 12 stages from embryo to larva. This analysis provided a useful resource for defining and manipulating specific neuronal subpopulations and for understanding the molecular mechanisms of vertebrate neurogenesis through the developmental mapping of the zebrafish brain [138]. To uncover the cellular dynamics that regulate neural maturation, Herring et al. conducted a single-cell sequencing analysis of human prefrontal cortex gene expression and chromatin accessibility from gestation to adulthood. The study revealed a clear and precise understanding of the underlying mechanisms of neural maturation. The comprehensive analysis defined the dynamic paths of every cell type, exposed vital gene expression reorganizations during the transition from prenatal to postnatal stages followed by ongoing reorganization in adulthood, and identified the regulatory network that governs cellular developmental programs, states, and functions [131].

In embryonic development, the single-cell transcriptome can aid researchers in exploring new mechanisms of development. Srivatsan et al. utilized sci-Space on developing mouse embryos, obtaining roughly 120,000 nuclei with their approximate spatial coordinates and complete transcriptomes. Thousands of genes displaying anatomical patterns of expression were identified, indicating a correlation between pseudotime and the migration patterns of differentiated neurons [139]. Mittnenzweig and colleagues developed a temporal model of mouse protoganglionic embryo formation based on the scRNA-seq. The study utilized data from 153 embryos that were individually sampled and sequenced over 36 h of embryonic development to construct a quantitative model for cell fate decisions [140].

In the field of plant development, Xia and colleagues demonstrated the single-cell spatial transcriptome mapping of Arabidopsis leaves using single-cell spatial enhanced resolution omics sequencing. They found a cell type-specific gene expression gradient from the main vein to the leaf margin, thus revealing diverse spatial developmental trajectories of vascular and guard cells [132]. The shoot apical meristem located in the stem enables the plant to produce innovative aerial root structures throughout its lifespan. Zhang et al. utilized single-cell RNA sequencing to chart the transcriptome-based developmental pathways and cell cycle continuity of Arabidopsis thaliana epidermal tissue, vascular tissue, and chloroplasts. Transcription factors and signatures of gene expression linked to cell fate decisions were inferred [141]. In a single-cell transcriptomic study of maize stem tips, Zhang et al. monitored changes in gene expression associated with cell differentiation. The ectopic expression of the *KN1* gene was discovered to accelerate cell differentiation and promote leaf base development in sheath maize [142].

In general, cellular dynamics change frequently during development, including cell fate, cell differentiation, and apoptosis. The application of single-cell transcriptomes to developmental studies of animal embryos and brain tissues has led to several important new discoveries. In plant development, single-cell transcriptomics has also revealed a variety of cellular transcriptional changes in rapidly growing areas such as leaves and roots. All of these discoveries have been attributed to the sensitivity and accuracy of this technology in detecting gene expression in single cells.

### 5.5. Cell Subpopulation Refinement and Rare Cell Type Identification

Hatscher et al. have identified a new subpopulation of dendritic cells, human type 2 conventional dendritic cells, which help to defend against invading viruses, bacteria or potentially lethal tumor cells by persistently stimulating some of the body’s immune cells, and the type 2 dendritic cell subset does not die upon antigen presentation [8]. For the first time, a subpopulation of astrocytes expressing the oxytocin receptor in the rodent brain was identified via scRNA-seq analysis. This group of cells mediates the anxiolytic and positive reinforcing effects of oxytocin in the central amygdala of both mice and rats. This finding defies the conventional viewpoint that oxytocin exclusively affects neurons and proposes that astrocytes are equally crucial in regulating emotional states [9]. Aizarani et al. have identified a scarce subpopulation of cholangiocytes that can form organoids. These precursor cells can differentiate into hepatocytes or cholangiocytes, and they may have a crucial function in liver regeneration [10]. Park and colleagues utilized single-cell gene expression to map the entire mouse kidney and identified three novel cellular subpopulations [11]. Their findings suggest that cells targeted to sodium/potassium balance can be transformed into cells targeted to acid/base balance. Undeniably, single-cell transcriptome sequencing is an incredibly valuable tool for researchers as it can detect cellular subpopulations, aiding in the exploration of new cellular functions. Currently, novel cell subpopulations are consistently being uncovered, and their novel functions are always surprising and expected.

### 5.6. Stem Cells Research

Single-cell sequencing technology can comprehensively analyze stem cell heterogeneity by identifying various types of differentiated cells at different stages of stem cell differentiation. It also enables the determination of marker genes for different subtypes of stem cells and the mapping of their developmental and differentiation trajectories.

To address the worldwide challenge of the insufficient durability of livestock stem cells during extended cycles and multiple gene editing, Zhi et al. conducted high-quality scRNA-seq mapping of porcine embryos on days 0–14 and established the porcine stem cell line with the highest number of passages globally. This achievement provides a critical basis for further researching the regulatory mechanism of porcine pluripotent stem cells [14]. Ayyaz et al. conducted an analysis of the mouse intestine through single-cell transcriptome sequencing. Their study identified a unique quiescent cell type, called resurrection stem cells, which can be activated through injury. These cells are characterized by a high expression of cohesin and are extremely rare during homeostatic conditions. However, they have the potential to give rise to all major intestinal cell types, including LGR5+CBCs [15]. After the targeted removal of LGR5+CBC cells or treatment with sodium dextrose sulfate causing damage to the gut, stem cells that have been resurrected undergo a short-lived proliferation dependent on YAP1. This results in the reconstruction of the LGR5+CBC compartment and, ultimately, the regeneration of a functional gut [15]. The application of scRNA-seq to stem cell research allows the resolution of stem cell characteristics that are easy to differentiate, identifies stem cell-specific markers, and further elucidates the direction of the differentiation of different stem cell types.

### 5.7. Applications in Microbiology

Rolling circle amplification represents the initial methodology reported for single-cell transcriptome analysis in prokaryotic cells [143,144]. Ribo-SPIA is capable of analyzing not only eukaryotic cells but also prokaryotic microorganisms. In 2011, Ribo-SPIA was first applied to synthesize and amplify cDNA from a single Aspergillus niger cell of eukaryotic microorganisms for single-cell transcriptome analysis [145]. The initial scRNA-seq analysis of prokaryotic cells was conducted in 2015, and the results indicated that ribo-SPIA starting sample sizes of less than 1 picogram were capable of detecting up to 98% of the expressed genes from single cells of the cyanobacterium Cyanobacteria collectivum. The method demonstrated high sensitivity and accuracy [146]. SPLiT-seq is a method for single-cell RNA sequencing in eukaryotes, while MicroSPLiT is a derivative of SPLiT-seq that is employed for the analysis of microbial communities in natural environments. Kuchina and colleagues sequenced the transcriptome of bacteria using microSPLiT [16]. This technique can be applied to both Gram-negative and Gram-positive bacteria, offering low-cost and high-throughput benefits. However, it relies on SPLiT-seq for intracellular amplification of the transcriptome and thus faces the same limitations as SPLiT-seq, including a restriction on the number of genes sequenced. Therefore, advancements of microbe-based single-cell transcriptome technology are still required.

### 5.8. Drug Resistance Research

The immune evasion and drug resistance of cancer cells are enormous challenges for many doctors. Here, we summarize the application of single-cell transcriptome technology to reveal the formation and changes of drug-resistant cells, which is helpful to avoid drug tolerance. Huang et al. conducted a comprehensive analysis of the developmental status map of human B-cell acute lymphoblastic leukemia through single-cell transcriptome analysis, which revealed a close correlation with sensitivity to the commonly used chemotherapy drug asparaginase. B-cell acute lymphoblastic leukemia patients exhibit significant heterogeneity in asparaginase reaction. By targeting BCL2, researchers found that venetoclax significantly enhances the efficacy of asparaginase in vitro and in vivo [147]. It has been reported that acquired resistance to tyrosine kinase inhibitors (TKIs) is inevitable in lung cancer carrying mutations in the epidermal growth factor receptor. Wang et al. observed that EOMES+CD8+ T cells exhibited a notable increase in TKI-resistant patients, which was found to be associated with a reduction in survival rates. The analysis of pseudotime and gene set variation by scRNA-seq suggests that the increase in EOMES+CD8+T cells may be due to T cell transformation and metabolic reprogramming. This suggests a correlation between the increase in the number of EOMES+CD8+ T cells and the development of acquired TKI resistance [148]. The drug resistance of trastuzumab represents a significant challenge in the treatment of human epidermal growth factor receptor 2 (HER2)-positive breast cancer. Du et al. identified a novel subset of fibroblasts (CAFs) enriched in trastuzumab-resistant tumor tissues using scRNA-seq. This subpopulation induces trastuzumab resistance in HER2+ state cancers by inhibiting NK cell-mediated ADCC immune response. Therefore, PDPN+CAFs may be a new therapeutic target to improve the sensitivity of HER2+ breast cancer to trastuzumab [149]. Glioblastoma is a highly heterogeneous brain tumor. Wu et al. conducted a transcriptome profiling study at the single-cell level, investigating the heterogeneity and resistance mechanisms of recurrent and drug-resistant glioblastoma multiforme (GBM) tumors. A preliminary identification of nine major cell clusters associated with recurrence and drug-resistant GBM has been made. In comparison to the initial GBM tissue, recurrent GBM tissue exhibits a reduction in the proportion of microglia. Furthermore, the O6-methylguanine DNA methyltransferase-related signaling pathway is activated in recurrent GBM. This provides new insights into the treatment strategies for recurrent glioblastoma [150]. The tumor immune microenvironment represents the primary factor contributing to the formation of drug resistance. Xue et al. conducted scRNA-seq analysis on 189 samples collected from one hundred twenty-four cancer patients and eight mice. Their findings indicated that a tumor-associated neutrophil (TAN) population, which was rich in myeloid cell subtypes, was associated with a poor prognosis. It has demonstrated that CCL4+TAN can recruit macrophages, while PD-L1+TAN can inhibit T cell cytotoxicity, thereby elucidating the pro-tumor phenotype of TAN [151].

### 5.9. Integration and Utilization of Single-Cell Datasets

Currently the integration and analysis of multiple platforms with multiple single-cell transcriptome sequencing data are beneficial for the in-depth mining and utilization of data, but there are still many problems and challenges. Stuart et al. developed a strategy to “anchor” different datasets together, which not only integrates single-cell sequencing data with different scRNA-seq technologies, but also integrates single-cell sequencing data with different modalities [78]. Theis et al. reported a single-cell population-level integration approach (scPoli), applying scPoli to the population-level mapping of lung cells and peripheral blood mononuclear cells, the latter of which consisted of 7.8 million cells from 2375 samples. scPoli is also applicable to single-cell sequencing analyses of transposase chromatin accessibility and cross-species datasets, providing an important tool for chromatin accessibility and comparative genomics insights [152].

Furthermore, the integration and standardization of disparate scRNA-seq datasets represents a significant challenge. The integration and standardization of scRNA-seq data are beset by a multitude of issues and obstacles, including batch effects, data sparsity, high dimensionality, biological heterogeneity, and the high demand for computing resources. Five data integration methods have been developed for scRNA-seq data: Harmony, Canonical Correlation Analysis (CCA), Reciprocal PCA (RPCA), Fast Mutual Nearest Neighbors (FastMNN), and Single-cell Variational Inference (scVI). The Harmony algorithm is highly efficient for large datasets, offering fast computation and low memory use, but its performance depends on the initial embedding quality [153]. Conversely, CCA is robust for identifying correlations in high-dimensional data but has high computational demands [78]. Similarly, RPCA maintains biological signals in noisy data through iterative processes but may have lower computational efficiency and be sensitive to initial parameters [154]. FastMNN excels in matching cells across batches but relies on neighboring cell quality [155]. scVI handles complex, heterogeneous data well but requires significant computational resources and optimization [156]. Each method has unique strengths and limitations. Therefore, the choice of method should consider factors such as data scale, noise levels, batch effect complexity, and available computational resources. For instance, Harmony and FastMNN are ideal for large-scale data integration, CCA and RPCA are advantageous for multi-batch data processing, and scVI excels in capturing complex nonlinear relationships and handling highly heterogeneous data.

## 6. Summary and Prospect

The emergence of single-cell transcriptome technology has made it possible to analyze specific characteristics of individual cells or cell populations, particularly playing a key role in areas such as cell development, tumor microenvironment, and single-cell mapping. Common platforms for single-cell transcriptome analysis currently include 10 × Genomics, BD Rhapsody, Fluidigm C1, and Bio-Rad, with the 10 × Genomics platform being the most prevalent due to its cost and throughput advantages. Although single-cell sequencing is capable of identifying and grouping the vast majority of cell types, it may not be applicable for some large-volume cells, such as adipocytes. At present, single-nucleus RNA sequencing (snRNA-seq) analysis can be considered for this type of cell. However, there is controversy over the consistency between the results of snRNA-seq and scRNA-seq. One potential avenue for further investigation is the possibility of separating the nucleus and cytoplasm for sequencing purposes. In the future, it is necessary to develop scRNA seq technology with greater compatibility.

As scRNA-seq technology has advanced, new methods have been developed. In recent years, the integration of single-cell transcriptomics with three-dimensional spatial analysis has led to the development of spatial single-cell transcriptome technologies such as eqFISH, MERFISH, and SCAN-seq2. However, challenges remain in terms of analyzing large areas and achieving high transcript coverage in spatial single-cell transcriptomics. High-throughput spatial transcriptome technologies at the single-cell level still require further development and optimization.

In terms of application, scRNA-seq technology has enhanced our biological understanding of embryonic cells [157,158], intracranial neurons [38,159], malignant tumor cells [160,161,162,163], and immune cells [164]. scRNA-seq and its derivatives help identify new markers, rare subpopulations, and evolutionary patterns, especially in brain development, human cell mapping, and cancer research. scRNA-seq is highly valuable for identifying biomarkers that guide clinical decision-making. It can dissect cellular heterogeneity within tumors or tissues, identifying distinct subpopulations with unique gene expression signatures that serve as biomarkers for diagnosis or prognosis. It detects rare cell types crucial in disease progression or treatment response. By analyzing individual cell expression profiles, scRNA-seq identifies biomarkers linked to drug sensitivity or resistance, predicting patient responses to therapies. It monitors treatment responses, revealing early signs of therapeutic resistance or efficacy and guiding treatment adjustments. scRNA-seq also discovers novel therapeutic targets by analyzing regulatory networks and pathways altered in diseases and profiling the immune microenvironment of tumors, which predicts responses to immunotherapy. Overall, scRNA-seq provides a comprehensive view of gene expression, enhancing clinical decision-making and enabling personalized treatments.

Despite limitations in sensitivity, scale, and accuracy, as well as challenges in spatial and temporal reconstruction, the exponential growth of scRNA-seq applications in basic and translational research is expected with continual improvements and standardization in experimental and analytical processes. In particular, the combination of scRNA-seq with other omics technologies, such as single-cell genomics and scATAC-seq, holds promise for elucidating gene regulatory mechanisms at the single-cell level. At present, there are technologies for the simultaneous sequencing of individual cells, including DBiT-seq [45], which can simultaneously detect mRNA and proteins, and MUSIC nucleic acid interactions analysis technology [165], which can detect multiple RNA and DNA expressions and interactions in single cells. scTrio-seq represents a combined profiling approach to examine the genome, methylome and transcriptome in cancer cells [104]. NEAT-seq is a technique that simultaneously profiles the abundance of intracellular proteins, chromatin accessibility, and the transcriptome in single cells [166]. It is anticipated that further analogous technologies will be developed in the future with the objective of achieving multi-omics within a single cell.

With the generation of various sequencing platforms and algorithms, as well as advancements and integration in multidisciplinary technologies, scRNA-seq technology is poised for new breakthroughs and further development. The future integration of single-cell multi-omics methods in laboratory and clinical settings is expected to have a greater impact.

## Figures and Tables

**Table 1:** Comparison of representative spatial transcriptome techniques.

Name	Method	Single-Cell Resolution	Analysis Area	Time Required	Advantage	Disadvantage
10 × Genomics Visium	Sequencing-based	~55 µm/spot	(6.5 mm × 6.5 mm) × 4	<1 h	Easy and convenient operation, short test time, high cell flux and cell capture efficiency, and good cell compatibility	Only analyzes RNA with poly A, high sample size requirements and non-full-length information
Stereo-seq	Sequencing-based	Close to single cell	10 mm × 10 mm (13 cm × 13 cm)	<1 h	Nanoscale resolution, high cell capture rate, panoramic field of view	Requires specific equipment and optimized sample handling
MERFISH	Imaging-based	≤100 nm/spot	Full slide (theoretically)	<24 h	High spatial resolution, large area imaging, high sensitivity, diverse applicability, multi-omics research	Specialized equipment required, high-cost, low detection efficacy
seqFISH	Imaging-based	Subcellular level	0.5 mm × 0.5 mm	2–3 days	Highly specific, limiting off-target effects	Expensive and time-consuming
DBiT-seq	Microfluidic chip Sequencing-based	~10 μm/pixel	Full slide (theoretically)	5–7 h	Simultaneous detection of mRNA and protein	Large inter batch differences, and low capture efficiency

## Data Availability

Not applicable.
